# Supplementary figures and images for: Comparative characteristics of oat doubled haploids and oat × maize addition lines: Anatomical features of the leaves, chlorophyll a fluorescence and yield parameters
Source: PLoS One. 2024 Apr 9;19(4):e0298072. doi: 10.1371/journal.pone.0298072 (PMC11003612; doi:10.1371/journal.pone.0298072)

A

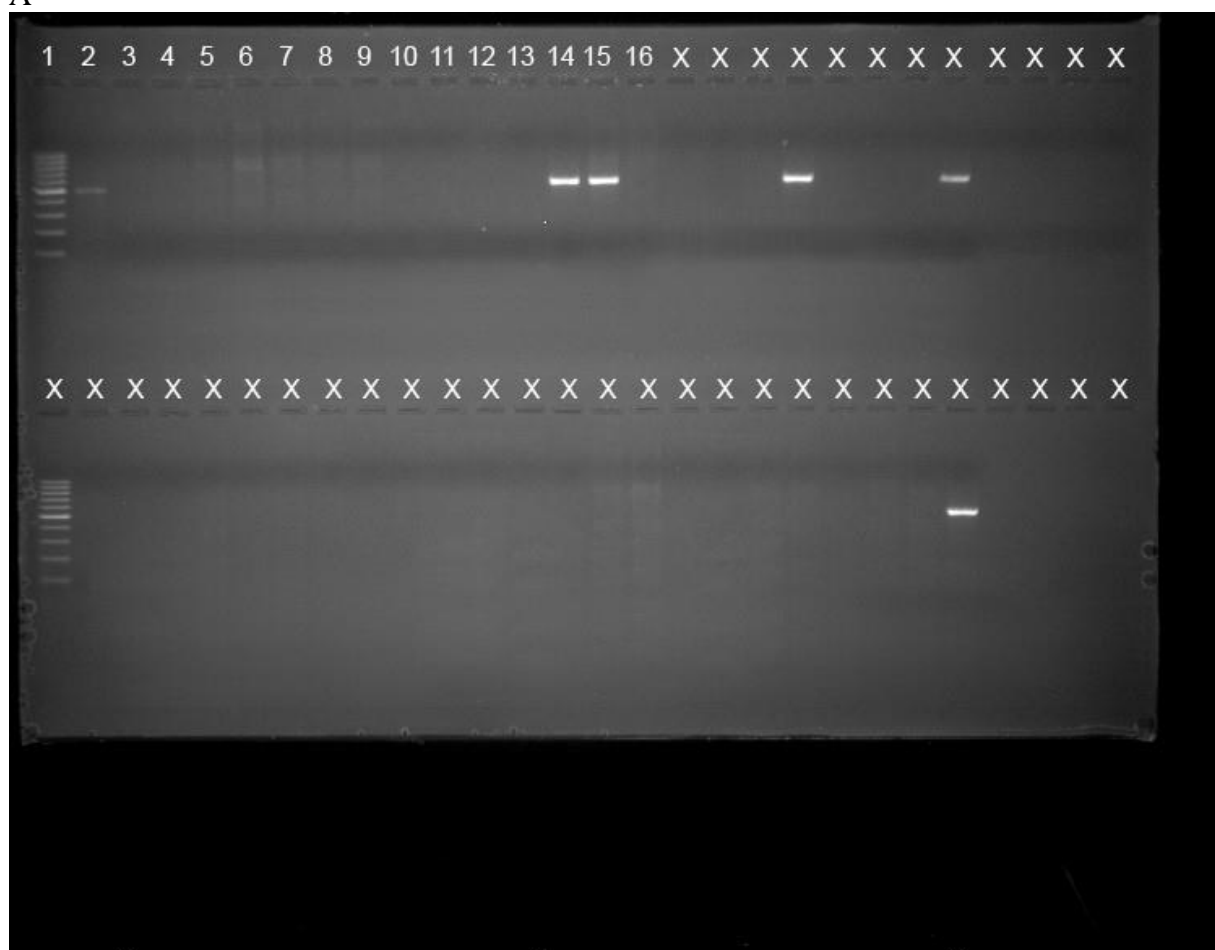

B

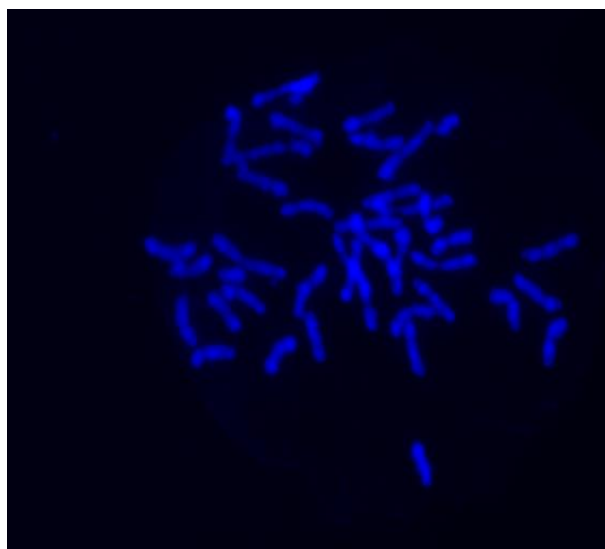

C

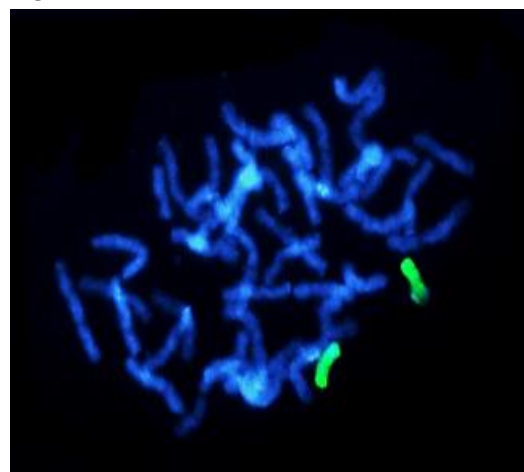

Supplement: S1 Raw image — Identification of maize (Zea maize L.) chromatin added to the oat (Avena sativa L.) genome by PCR and genomic in situ hybridization (GISH). (A) The agarose gel with bands representing DNA fragments Grande 1 (500 bp) specific for maize; path 1 –marker leader, path 2 –maize cv. Waza, path 3 –oat cv. Bingo, paths 4–13 DH lines of oat, paths 14–15 OMA lines, path 16 DH line of oat, X—lanes not included in the final figure. (B) Chromosomes of doubled haploid line (DH I), (C) Chromosomes of oat × maize addition line (OMA I). Blue fluorescence: DAPI, green fluorescence: maize genomic DNA (gDNA). (PDF) [file pone.0298072.s001.pdf]
