# Supplementary material for: Comparative characteristics of oat doubled haploids and oat × maize addition lines: Anatomical features of the leaves, chlorophyll a fluorescence and yield parameters
Source: PLoS One. 2024 Apr 9;19(4):e0298072. doi: 10.1371/journal.pone.0298072 (PMC11003612; doi:10.1371/journal.pone.0298072)

PLOS ONE

Comparative characteristics of oat doubled haploids and oat × maize addition lines: anatomical features of the leaves, chlorophyll a fluorescence and yield parameters

--Manuscript Draft--

**Manuscript Number:**	PONE-D-23-25096
**Article Type:**	Research Article
**Full Title:**	Comparative characteristics of oat doubled haploids and oat × maize addition lines: anatomical features of the leaves, chlorophyll a fluorescence and yield parameters
**Short Title:**	Characteristics of oat doubled haploids and oat × maize addition lines
**Corresponding Author:**	Marzena Warchoł<br>Polish Academy of Sciences The F. Górski of Plant Physiology<br>Kraków, POLAND
**Keywords:**	
**Abstract:**	As a result of oat (Avena sativa L.) × maize (Zea mays L.) crossing, maize chromosomes may not be completely eliminated at the early stages of embryogenesis, leading to the oat × maize addition (OMA) lines development. Introgression of maize chromosomes into oat genome can cause morphological and physiological modifications. The aim of the research was to evaluate the leaves' anatomy, chlorophyll a fluorescence, and yield parameter of oat doubled haploid (DH) and OMA lines obtained by oat × maize crossing. The present study examined two DH and two disomic OMA lines and revealed that they differ significantly in the majority of studied traits, apart from: the number of cells of the outer bundle sheath; light energy absorption; excitation energy trapped in PSII reaction centers; and energy dissipated from PSII. The OMA II line was characterized by larger size of single cells in the outer bundle sheath and greater number of seeds per plant among tested lines.
**Order of Authors:**	Marzena Warchoł
	Katarzyna Juzoń-Sikora
	Dragana Rančić
	Ilinka Pećinar
	Tomasz Warzecha
	Dominika Idziak-Helmcke
	Kamila Laskoś
	Ilona Czyczyło-Mysza
	Kinga Dziurka
	Edyta Skrzypek
**Additional Information:**	
**Question**	**Response**
**Financial Disclosure**<br><br>Enter a financial disclosure statement that describes the sources of funding for the work included in this submission. Review the submission guidelines for detailed requirements. View published research articles from *PLOS ONE* for specific examples.	This research was funded by the F. Górski Institute of Plant Physiology Polish Academy of Sciences, project No T.1 ZB.2/2019, and by the Ministry of Science, Technological Development, and Innovation of the Republic of Serbia No 451-03-47/2023-01/200116. The oat DH and OMA lines were produced under the National Centre for Research and Development Poland grant no. PBS3/B8/17/2015. The funders did not and will not have a role in study design, data collection and analysis, decision to publish, or preparation of the manuscript.

Powered by Editorial Manager® and ProduXion Manager® from Aries Systems Corporation

This statement is required for submission and **will appear in the published article** if the submission is accepted. Please make sure it is accurate.<br><br>**Unfunded studies**<br>Enter: *The author(s) received no specific funding for this work.*<br><br>**Funded studies**<br>Enter a statement with the following details:<br>• Initials of the authors who received each award<br>• Grant numbers awarded to each author<br>• The full name of each funder<br>• URL of each funder website<br>• Did the sponsors or funders play any role in the study design, data collection and analysis, decision to publish, or preparation of the manuscript?<br>• **NO** - Include this sentence at the end of your statement: *The funders had no role in study design, data collection and analysis, decision to publish, or preparation of the manuscript.*<br>• **YES** - Specify the role(s) played.<br><br>* typeset	
**Competing Interests**<br><br>Use the instructions below to enter a competing interest statement for this submission. On behalf of all authors, disclose any competing interests that could be perceived to bias this work—acknowledging all financial support and any other relevant financial or non-financial competing interests.<br><br>This statement is required for submission and **will appear in the published article** if the submission is accepted. Please make sure it is accurate and that any funding sources listed in your Funding Information later in the submission form are also declared in your Financial Disclosure statement.<br><br>View published research articles from *PLOS ONE* for specific examples.	The authors have declared that no competing interests exist.

**NO authors have competing interests**<br><br>Enter: *The authors have declared that no competing interests exist.*<br><br>**Authors with competing interests**<br><br>Enter competing interest details beginning with this statement:<br><br>*I have read the journal's policy and the authors of this manuscript have the following competing interests: [insert competing interests here]*<br><br>* typeset	
**Ethics Statement**<br><br>Enter an ethics statement for this submission. This statement is required if the study involved:<br><br>• Human participants<br>• Human specimens or tissue<br>• Vertebrate animals or cephalopods<br>• Vertebrate embryos or tissues<br>• Field research<br><br>Write "N/A" if the submission does not require an ethics statement.<br><br>General guidance is provided below. Consult the submission guidelines for detailed instructions. **Make sure that all information entered here is included in the Methods section of the manuscript.**	N/A

**Format for specific study types**<br><br>**Human Subject Research (involving human participants and/or tissue)**<br>• Give the name of the institutional review board or ethics committee that approved the study<br>• Include the approval number and/or a statement indicating approval of this research<br>• Indicate the form of consent obtained (written/oral) or the reason that consent was not obtained (e.g. the data were analyzed anonymously)<br><br>**Animal Research (involving vertebrate animals, embryos or tissues)**<br>• Provide the name of the Institutional Animal Care and Use Committee (IACUC) or other relevant ethics board that reviewed the study protocol, and indicate whether they approved this research or granted a formal waiver of ethical approval<br>• Include an approval number if one was obtained<br>• If the study involved *non-human primates*, add *additional details* about animal welfare and steps taken to ameliorate suffering<br>• If anesthesia, euthanasia, or any kind of animal sacrifice is part of the study, include briefly which substances and/or methods were applied<br><br>**Field Research**<br><br>Include the following details if this study involves the collection of plant, animal, or other materials from a natural setting:<br>• Field permit number<br>• Name of the institution or relevant body that granted permission	
**Data Availability**<br><br>Authors are required to make all data underlying the findings described fully available, without restriction, and from the time of publication. PLOS allows rare exceptions to address legal and ethical concerns. See the PLOS Data Policy and FAQ for detailed information.	Yes - all data are fully available without restriction

A Data Availability Statement describing where the data can be found is required at submission. Your answers to this question constitute the Data Availability Statement and **will be published in the article**, if accepted.<br><br>**Important:** Stating 'data available on request from the author' is not sufficient. If your data are only available upon request, select 'No' for the first question and explain your exceptional situation in the text box.<br><br>Do the authors confirm that all data underlying the findings described in their manuscript are fully available without restriction?	
**Describe where the data may be found in full sentences. If you are copying our sample text, replace any instances of XXX with the appropriate details.**<br><br>• If the data are **held or will be held in a public repository**, include URLs, accession numbers or DOIs. If this information will only be available after acceptance, indicate this by ticking the box below. For example: *All XXX files are available from the XXX database (accession number(s) XXX, XXX.).*<br>• If the data are all contained **within the manuscript and/or Supporting Information files**, enter the following: *All relevant data are within the manuscript and its Supporting Information files.*<br>• If neither of these applies but you are able to provide **details of access elsewhere**, with or without limitations, please do so. For example:<br><br>*Data cannot be shared publicly because of [XXX]. Data are available from the XXX Institutional Data Access / Ethics Committee (contact via XXX) for researchers who meet the criteria for access to confidential data.*<br><br>*The data underlying the results presented in the study are available from (include the name of the third party*	All the data are contained within the manuscript.

and contact information or URL).<br>• This text is appropriate if the data are owned by a third party and authors do not have permission to share the data.<br><br>* typeset	
Additional data availability information:	

Powered by Editorial Manager® and ProduXion Manager® from Aries Systems Corporation

Manuscript

Click here to access/download;Manuscript;Manuscript_07.08.23.docx

Comparative characteristics of oat doubled haploids and oat × maize addition lines: anatomical features of the leaves, chlorophyll *a* fluorescence and yield parameters

Marzena Warchoł[1*], Katarzyna Juzoń-Sikora[1], Dragana Rančić[2], Ilinka Pećinar[2], Tomasz Warzecha[3], Dominika Idziak-Helmcke[4], Kamila Laskoś[1], Ilona Czyczyło-Mysza[1], Kinga Dziurka[1], Edyta Skrzypek[1]

[1]*The Franciszek Górski* Institute of Plant Physiology, Polish Academy of Sciences, Kraków, Poland

[2]Faculty of Agriculture, University of Belgrade, Belgrade, Serbia

[3]Department of Plant Breeding, Physiology and Seed Science, University of Agriculture in Kraków, Kraków, Poland

[4]Institute of Biology, Biotechnology, and Environmental Protection, University of Silesia in Katowice, Katowice, Poland

*Correspondence: m.warchol@ifr-pan.edu.pl (MW)

Abstract

As a result of oat (*Avena sativa* L.) × maize (*Zea mays* L.) crossing, maize chromosomes may not be completely eliminated at the early stages of embryogenesis, leading to the oat × maize addition (OMA) lines development. Introgression of maize chromosomes into oat genome can cause morphological and physiological modifications. The aim of the research was to evaluate the leaves' anatomy, chlorophyll *a* fluorescence, and yield parameter of oat doubled haploid (DH) and OMA lines obtained by oat × maize crossing. The present study examined two DH and two disomic OMA lines and revealed that they differ significantly in the majority of studied traits, apart from: the number of cells of the outer bundle sheath; light energy absorption; excitation energy trapped in PSII reaction centers; and energy dissipated from PSII. The OMA II line was characterized by larger size of single cells in the outer bundle sheath and greater number of seeds per plant among tested lines.

Introduction

As a result of distant crossings of cereals, hybrid embryos of wheat, oat, and barley are formed, and during subsequent divisions of the nucleus, the paternal chromosomes are eliminated, which in turn leads to the formation of haploid embryos with only the mother's genetic material [1–3]. However, sometimes this elimination of paternal chromosomes does not occur properly and fragments or complete donor chromosomes are incorporated into the mother's genome. Most often it takes place in the crosses of plants belonging to the two subfamilies Pooideae and Panicoideae, within the family Poaceae. Extra chromosomes of maize (*Zea mays* L.) or pearl millet (*Pennisetum glaucum* (L.) R. Br.) have been observed in both haploids of wheat [1,4] and oat [5]. Studying the causes of this phenomenon, Mochida et al. [6] found incomplete attachment of the spindle to maize centromeres, and Ishii et al. [7] identified the breakage of pearl millet chromosomes. The presence of stable maize chromosomes in the

oat genome was first described by Riera-Lizarazu et al. [5], and a few years later Kynast et al. [8] called them oat × maize addition (OMA) lines. Since then maize chromosome-addition lines of oat represent unique tools for researching the maize gene expression in oat, the structure of maize chromatin, or intergeneric translocations [5]. These lines have ~~been used also~~ for physical mapping of the maize genome [9], centromere-specific histone studies CENH3 [10], or the introduction of maize traits into oat, such as disease resistance [11]. The OMA lines, which are hybrids of C_3 and C_4 plants, were used by Kowles et al. [12] not only to better understood the C_4 photosynthesis mechanism but also for studying the possibilities of the transfer of C_4 photosynthesis from maize to oat. To analyze the genetics of the C_4 pathway in maize and to identify the chromosomes and chromosome areas that are crucial for C_4 photosynthesis, the expression of maize phosphoenolpyruvate carboxylase (*PEPc*) and pyruvate orthophosphate dikinase (*PPDK*) genes at the mRNA level in appropriate OMA lines was studied [12]. PEPc enzyme activity was discovered to be 5.0 times higher in the OMA9 line, and PPDK enzyme activity to be 17.6 times higher in the OMA6 line than that of the oat background line.

Dengler and Nelson [13] and Sage et al. [14] ~~have~~ reported 22 versions of the two-celled Kranz anatomy, and according to these authors, five varieties of the Kranz anatomy were found in grasses that contained two sheaths encircling each vascular bundle. The parenchymal bundle sheath (PBS) surrounds the inner sheath, which is known as the mestome sheath and possesses endodermal features. The PBS is where Rubisco is located in each of the Kranz variations and is used to fix CO_2. To our knowledge, only Tolley et al. [15] conducted research on the anatomical structure of the leaves of the OMA lines, and showed that two key traits of C_4 photosynthesis, increased PBS cell size and reduced vein spacing in the C_3 leaf, can be changed by the introduction of maize chromosomes into the oat genome. Moreover, these authors studied lipid deposition in oat vascular bundle sheath cells, and indicated that chromosome 3

likely contains loci that can control PBS cell wall lipid deposition but not the entire suberin deposition pathway.

Since C_4 plants grow faster than C_3 plants and also require less water, plant biologists would like to introduce certain C_4 traits into C_3 crop plants including tolerance to rapidly changing environmental factors [16]. The characteristics of the OMA line in conditions of soil drought were described by Juzoń et al. [17], who found that OMA lines predominate the Bingo cultivar in terms of photosynthetic apparatus efficiency both in optimal and adverse conditions. Recently, Warzecha et al. [18] stated that the effect of drought stress was significant in all considered parameters of chlorophyll *a* fluorescence kinetics (PCF). The most affected indices of the PSII photosystem were the overall performance index of PSII photochemistry (PI) and the Area – pool size of the electron acceptors from PSII. Because drought is considered as one of the most important factors limiting the productivity of crops, transgenic methods to modify C_4 photosynthetic enzymes in an effort to convert C_3 plants into C_4 plants is a current field of research. In order to increase yield potential, engineering C_4 photosynthesis into rice is being attempted by the international C_4 Rice Consortium [19].

Many of the OMA lines display specific phenotypes, which indicates that maize genes are likely expressed and capable of altering the phenotype of oat plants [20]. These findings were corroborated by the work of Skrzypek et al. [21], in which the OMA lines differed from the doubled haploid (DH) lines. Moreover, the earlier study of Kynast et al. [8] revealed that the presence of maize chromosomes in oat genome caused an erect leaf phenotype, or the appearance of leaf blades with necrotic and chlorotic spots compared to *cv*. Sun II.

The aim of our study was to evaluate how the presence of maize chromosomes changes the anatomical parameters of the leaves and functioning of photosynthetic apparatus of the disomic OMA lines compared to the DH lines and Bingo cultivar. We assessed also yield components for all lines. This research extends knowledge about hybrids in the context of more targeted

efforts to improve crop productivity. To our knowledge, for the first time, the OMA and DH lines produced from the same parental crosses were compared in terms of leaf anatomical features, taking into account the parameters of chlorophyll *a* fluorescence kinetics and yielding. We would like to show that the differences between the tested plants may result not only from the presence or absence of maize chromosomes in the oat genome, but are also the result of parental background.

Results

Identification of oat × maize hybrids

The presence of maize chromosome introgression in OMA I and OMA II, which was detected by PCR using primers specific for ubiquitous highly repetitive maize retrotransposon Grande-1 (Fig 1A), was further validated by GISH using maize gDNA as a probe. The experiments did not require using unlabeled oat genomic DNA in order to block non-specific hybridization between oat and maize due to high phylogenetic distance between those species. In both OMA lines GISH clearly detected the presence of two maize chromosomes added to a full set of oat chromosomes. In contrast, DH I and DH II both possessed only a clear set of *Avena sativa* L. chromosomes (Table 1, Fig 1B). The analysis with maize chromosome-specific SSR markers revealed that the OMA I line was a disomic characterized by the presence of a pair of maize chromosome 5 (2n = 44 (42 + Zm5”)) (Fig 1C), whereas line OMA II was a double monosomic with maize chromosomes 3 and 8 (2n = 44 (42 + Zm3’ + Zm8’)).

Fig 1. Identification of maize (*Zea maize* L.) chromatin added to the oat (*Avena sativa* L.) genome by PCR and genomic *in situ* hybridization (GISH). (A) The agarose gel with bands representing DNA fragments *Grande 1* (500 bp) specific for maize; path 1 – marker leader, path 2 – maize cv. Waza, path 3 – oat cv. Bingo, paths 4-13 DH lines of oat, paths 14-15 OMA lines, path 16 DH line of oat (B) Chromosomes of doubled haploid line (DH I), (C)

Chromosomes of oat × maize addition line (OMA I). Blue fluorescence: DAPI, green fluorescence: maize genomic DNA (gDNA).

Table 1. Characteristics of the plant material used in the study.

**Line/cultivar**	**Origin**	**No. of maize chromosome added to oat genome**	**Chromosome ID**
DH I	Flamingstern × Bingo	-	-
OMA I	Flamingstern × Bingo	2	Zm5"
DH II	STH 9787(b) × Bingo	-	-
OMA II	STH 9787(b) × Bingo	2	Zm3' + Zm8'
cv. Bingo	-	-	-

Anatomical analysis of the DH and OMA leaves

An analysis of the variance showed significant differences between tested oat plants in terms of all the tested anatomical features (Table 2). In order to investigate whether the presence of individual maize chromosomes increased the number of vascular bundles in oat, distances between the midpoints of adjacent vascular bundles were measured in transverse sections of leaves from DH and OMA lines and oat cv. Bingo (Fig 2A – E). In the DH I and OMA I lines, which were both developed from Flamingstern × Bingo crosses, the mean distances between adjacent vascular bundles were approximately 400 µm and were bigger than in the DH II and OMA II lines (STH 9787(b) × Bingo) and in cv. Bingo (341 µm, 353 µm, and 334 µm, respectively) (Table 3). The DH I line was characterized by having the largest mesophyll area among all the tested oat plants and amounted to 86,791.12 µm^2, while the OMA I line and the cv. Bingo had similar values of the examined parameter (75,881.07 µm^2; 70,159.06 µm^2,

respectively). We found that the distance between the two bundles was affected by the germplasm used in the crosses and was not dependent on maize chromosomal introgression. Although there were no statistically significant differences in the number of cells of the outer bundle sheath, the entire area of the bundle sheath differed among the tested oat plants from the largest in OMA I and DH I (6670.36 µm², 6062.36 µm², respectively), to the smallest in OMA II and cv. Bingo (3711.20 µm², 3972.04 µm², respectively). In this study, attention was paid to the size of the cells forming the bundle sheath. The biggest size of a single cell in the outer bundle sheath, more than 500 µm^2, was noted in DH I and OMA I lines (Fig 2F).

Fig 2. Cross sections of oat (*Avena sativa* L.) leaves made by using the IM 1000 software package: (A) doubled haploid (DH I); (B) oat × maize addition line (OMA I); (C) doubled haploid (DH II); (D) oat × maize addition line (OMA II); (E) oat *cv.* Bingo; (F) vascular bundle of oat × maize addition line (OMA II); LVB – large vascular bundle, SVB – small vascular bundle, C – chlorenchyma, BSC – bundle sheath cells, S – sclerenchyma, E – epidermis.

Table 2. *F*-statistic from one-way analysis of variance for leaf anatomical traits of DH and OMA lines and *cv*. Bingo.

**Source of variation**	**Genotype**
Degrees of freedom	4 (k - 1)
Distance between two bundles [µm]	6.351***
Area of mesophyll between two bundles [µm^2]	19.260***
Number of cells of inner bundle sheath	3.343**
Area of inner bundle sheath [µm^2]	4.136**
Average size of single cell in inner bundle sheath [µm^2]	4.753**
Number of cells of outer bundle sheath	0.776^{ns}
Area of outer bundle sheath [µm^2]	22.018***
Average size of single cell in outer bundle sheath [µm^2]	25.809***

* $p \leq 0.05$, ** $p \leq 0.01$, *** $p \leq 0.001$, ns - not significant, k - number of genotypes

Table 3. Leaf anatomical traits of DH and OMA lines and *cv.* Bingo. The mean values (n = 5) ± SE marked with different letters are significantly different at $p \leq 0.05$ according to the Duncan's multiple test.

Line/ cultivar	Distance between two small bundles [μm]	Area of mesophyll between two bundles [μm²]	Number of cells of inner bundle sheath	Area of inner bundle sheath [μm²]	Average size of single cell in inner bundle sheath [μm²]	Number of cells of outer bundle sheath	Area of outer bundle sheath [μm²]	Average size of single cell in outer bundle sheath [μm²]
DH I	427.81 a	86791.12 a	11.70 b	1698.78 a	126.90 a	11.60 a	6062.36 a	542.96 a
OMA I	407.19 a	75881.07 b	12.60 ab	1735.74 a	124.37 a	12.10 a	6670.36 a	548.40 a
DH II	341.25 b	61126.58 c	13.40 a	1356.16 ab	115.27 ab	11.80 a	3711.20 c	266.79 c
OMA II	353.49 b	58968.41 c	13.80 a	1338.22 ab	95.30 bc	12.30 a	4843.76 b	401.40 b
cv. Bingo	334.32 b	70159.06 b	13.80 a	1120.56 b	88.35 c	11.50 a	3972.04 c	346.24 b

Characteristics of the photosynthetic apparatus of DH and OMA lines

Selected parameters of chlorophyll *a* fluorescence (PCF) were chosen in order to compare the functioning of photosynthetic apparatuses of the DH and OMA lines. The analysis of variance presented in Table 4 showed significant differences between tested oat plants in terms of Fv/Fm (maximum photochemical efficiency of PSII), Area (area over the chlorophyll *a* fluorescence induction curve), PI (overall performance index of PSII photochemistry) ET_0/CS (energy used for electron transport) and RC/CS_0 (number of active reaction centers in the excited leaf fragment) as opposed to ABS/CS (light energy absorption), TR_0/CS (excitation

energy trapped in PSII reaction centers), and DI_0/CS (energy dissipated from PSII). Data presented in Fig 3 showed that the OMA I line usually achieved the highest PCF indices apart from energy dissipation from PSII (DI_0/CS) compared to the other oat plants. The individuals from this line were characterized by maximum photochemical efficiency of PSII (Fv/Fm) and the biggest area over the chlorophyll a fluorescence induction curve (Area), which corresponded to the size of the reduced plastoquinone pool. Also, the overall performance index of PSII photochemistry (PI) was much higher than in other plants. The OMA I line plants used more energy for electron transport (ET_0/CS) but they were not the only ones with a high number of active reaction centers in the excited leaf fragment (RC/CS_0). The maximum photochemical efficiency of PSII was achieved by the OMA I line although the values of this parameter did not differ significantly in DH I and cv. Bingo. Photosynthetic apparatuses of all lines showed the same level of light energy absorption (ABS/CS), as well as excitation energy trapped in PSII reaction centers (TR_0/CS).

Fig 3. Chlorophyll *a* fluorescence kinetics parameters of the DH and OMA lines and cv. Bingo leaves. (A) Fv/Fm - maximum photochemical efficiency of PSII; (B) Area - area over the chlorophyll a fluorescence induction curve; (C) PI - overall performance index of PSII photochemistry; (D) ABS/CS - light energy absorption; (E) TR_0/CS - excitation energy trapped in PSII reaction centers; (F) ET_0/CS - energy used for electron transport; (G) DI_0/CS - energy dissipated from PSII; (H) RC/CS_0 - number of active reaction centers in the excited leaf fragment. The mean values (n = 5) ± SE marked with different letters are significantly different at $p \leq 0.05$ according to the Duncan's multiple test.

Table 4. *F*-statistic from one-way analysis of variance for chlorophyll *a* fluorescence kinetics parameters of DH and OMA lines and *cv*. Bingo. Fv/Fm - maximum photochemical efficiency of PSII; Area - area over the chlorophyll a fluorescence induction curve; PI - overall performance index of PSII photochemistry; ABS/CS - light energy absorption; TR_0/CS -

excitation energy trapped in PSII reaction centers; ET_0/CS - energy used for electron transport; DI_0/CS - energy dissipated from PSII; RC/CS_0 - number of active reaction centers.

Source of variation	Genotype
Degrees of freedom	4 (k - 1)
Fv/Fm	5.611^{**}
Area	6.461^{***}
P.I.	4.390^{**}
ABS/CS	0.196 ns
TR_0/CS	0.610 ns
ET_0/CS	3.137^{*}
DI_0/CS	2.695 ns
RC/CS_0	2.975^{***}

$^{*}\ p \leq 0.05$, $^{**}\ p \leq 0.01$, $^{***}\ p \leq 0.001$, ns - not significant, k - number of genotypes

Morphology and yield components of DH and OMA lines

An analysis of the variance revealed significant differences between tested oat plants in terms of all morphological traits and yield components (Table 5). The tested lines showed differences in flag leaf morphology, with the longest flag leaf observed in *cv*. Bingo (25.67 cm), and the shortest in OMA I (14.33 cm) (Fig 4). Despite this, the flag leaf of the latter line was the widest (9.33 mm). The OMA I line also had the tallest main shoot and the longest panicle, while those morphological traits were the smallest in the DH II and OMA II lines. The highest production of lateral shoots (9.00) as well as the number (262.67) and weight (16.49 g) of seeds per plant was found in *cv*. Bingo. On the other hand, no significant differences regarding the above mentioned yield components were observed between all tested lines. The results concerning TGW were particularly notable. The highest value of the TGW was recorded for

cv. Bingo and for the OMA I line (62.50 g; 53.10 g, respectively). Additionally, OMA I produced the smallest number of seeds per plant (0.99) among all tested lines.

Fig 4. The yield components of the DH and OMA lines and *cv*. Bingo: (A) flag leaf length [cm]; (B) flag leaf width [mm]; (C) height of the main shoot [cm]; (D) panicle length [cm]; (E) number of shoots; (F) number of seeds per plant; (G) weight of seeds per plant [g]; (H) thousand grain weight (TGW) [g]. The mean values (n = 5) ± SE marked with different letters are significantly different at p ≤ 0.05 according to the Duncan's multiple test.

Table 5. *F*-statistic from one-way analysis of variance for morphological traits and yield components of DH and OMA lines and *cv*. Bingo.

**Source of variation**	**Genotype**
Degrees of freedom	4 (k - 1)
Flag leaf length [cm]	3.852^{*}
Flag leaf width [mm]	9.921^{***}
Height of main shoot [cm]	51.924^{***}
Panicle length [cm]	22.127^{***}
Number of shoots	9.833^{***}
Number of seeds/plant	17.120^{***}
Weight of seeds/plant [g]	19.350^{***}
Thousand grain weight (TGW) [g]	22.229^{***}

* $p \leq 0.05$, ** $p \leq 0.01$, *** $p \leq 0.001$, ns - not significant, k - number of genotypes

Discussion

Interspecific plant crossings may omit fertilization obstacles and produce allopolyploids or haploids. Allopolyploids combine the genomes of both parental gametes, whereas haploids delete one parental genome upon fertilization. Chromosome addition lines, which fall between

allopolyploids and haploids in terms of genome interaction, can be produced through certain uncommon hybridization occurrences [22]. In interspecific crossings, chromosome addition lines are typically kept and they include a full set of uniparental chromosomes along with one or more pairs of chromosomes that were stably inherited from the distinct parent [23]. Oat × maize addition (OMA) lines described by Riera-Lizarazu et al. [5] were created through a sexual cross between oat (*Avena sativa* L., 2n = 6x = 42) and maize (*Zea mays* L., 2n = 20), which resulted in adding specific maize chromosomes to the entire oat genome. In the oat genome background, a single maize chromosome makes up approximately 2% of the total nuclear DNA [24]. In general, hybrids with one or two maize chromosomes resemble oat plants at every stage of growth, making it a challenge to determine how many maize genes are expressed or silenced. Recently, Dong et al. [25] discovered that more than 70% of the alien maize chromosomes' genes kept their original pattern of expression or transcription in the oat genomic environment. They hypothesized that a local cis region in the foreign maize chromosome may be primarily responsible for controlling the maize genes preserving the original transcription in OMA lines. Just two studies, as far as we are aware, looked at chromosomal-wide gene expression in chromosome addition lines [25,26].

The suppression of the C_4 pathway in hybrids between closely related C_3 and C_4 species has been documented by Brown and Bouton [27]. As researchers have shown, even the presence of a full genome complement fails to activate a C_4-like state in C_3–C_4 hybrids, which is why it is unlikely that an individual chromosome or chromosomes can initiate the C_4 program within the C_3 context. However, Kowles et al. [12] indicate that OMA lines possess entire chromosomes that contain maize transcription factors and promoters rather than just individual genes, which theoretically might lead to a rise in the activity of C_4 enzymes. Therefore, it seems reasonable to conduct research on the OMA lines that have the potential to help clarify the transfer of C_4 photosynthesis from maize to oat. The individual OMA lines were used to dissect the genetics

of the C_4 pathway of maize and to identify maize chromosomes and chromosome regions that are important in C_4 photosynthesis [12]. While the mono- and dicotyledonous C_4 lineages' leaf design can vary significantly, all C_4 plants have a wreath-like arrangement of mesophyll and bundle sheath cells around the vascular bundles called the Kranz anatomy [13]. The Kranz anatomy is accompanied by a dense vein spacing, which results in a reduced volume of mesophyll relative to bundle sheath tissue that in C_4 grasses may reach a ratio of nearly one to one. Because the close vein spacing in C_4 species appears to be mostly due to changes in the patterning of the minor and not the major vascular bundle [28], in our study after measuring the distance between the two small bundles, we did not observe a denser system of vascular bundle in either OMA line. However, Tolley et al. [15] showed that maize chromosomes are capable of altering vascular bundle spacing in C_3 leaves, but only one of two OMA lines in that study showed decreasing vascular bundle compared with the wild type. Maize chromosome 9 was common to both tested OMA lines, but they differed in their parental backgrounds, and probably the loci on maize chromosome 9 encoding this trait were silenced, depending on their interaction with the oat genome. Tolley et al. [15] reported that the lines possessing maize chromosome 1, 2, 3, 7, 8, or 9 had significantly larger PBS areas than either of the oat parental lines. This suggests that these chromosomal additions independently specified larger PBS cells. In our study, the area of the outer bundle sheath was the largest in line OMA I, which is in accordance with the above-cited studies, if it wasn't for that in line DH I (produced from the same crossing) this area was comparable with OMA I line. However, it is worth emphasizing that the OMA II line was characterized by a larger area of the outer bundle sheath not only in relation to the DH II line, but also to cv. Bingo. Overall, Dengler et al. [29] found that the pattern of variation in leaf blade anatomy is complex, reflecting correlations with both taxonomic group and photosynthetic type, and no new analytical tools have been developed to distinguish one biochemical type from another a priori.

The morphological observations of the examined plants revealed several differences within the DH and OMA lines. The OMA I line had taller main shoots, longer panicles, and shorter and wider flag leaf compared to the DH I line. Kynast et al. [8] also observed the differences in hybrid morphology, and their OMA plants either produced necrotic and chlorotic patches on the leaf blades or were characterized by premature senescence that caused yellowing and drying of shoots. Similar findings were made by Riera-Lizarazu et al. [5], who noted that DH oat plants were more robust and viable than plants with maize chromosomes. What is more, Juzoń et al. [17] described phenotypic differences within the OMA plants having four maize chromosomes and obtained from crossing the same parents. Among them there were oat-like plants with tall shoots that formed panicles and grass-like plants that did not form panicles. These modifications of phenotypes could have resulted from one of two causes: the interaction between the oat and maize transcriptomes might have changed the expression of phenotype-linked oat genes; or some ectopic expression of phenotype-linked maize genes might have taken place within the genomic background of oat. In our research, we did not observe symptoms similar to those reported by other researchers; thus, the second hypothesis seems more plausible. Additionally, Dong et al. [25] stated that gain-of-function abnormalities are uncommon and that transcriptome analysis did not reveal any apparent enhanced expression of phenotype-related genes. Even though the oat genome has not yet been sequenced, further research on the impact of alien maize chromosomes on the oat transcriptome in OMA lines may provide information about the mechanisms behind the OMA phenotypes.

Interest in introducing the C_4 route into C_3 crops to boost yield potential is dictated by the C_4 plants higher productivity [14]. Due to higher photosynthetically active radiation-use efficiency, species such as maize, sorghum, and sugarcane can have 50% higher yields than C_3 species [30,31]. Ways to increase the yield potential include improving photosynthesis and elevating photosynthetic utilization. It is worth stressing that OMA lines were characterized by

higher values of most of the analyzed PCF, which may confirm more efficient functioning of their photosynthetic apparatus compared to the DH lines and even the *cv*. Bingo. Similar observations were reported by Juzoń et al. [17], where among the examined OMA lines, the line with two retained maize chromosomes (2n = 44 (42 + Zm5″)) showed higher values for most parameters of chlorophyll a fluorescence. The greatest difference was observed in the Area, and this line showed almost two times higher values of this parameter. The authors also emphasized that not only the number of retained maize chromosomes but also the identity of introduced maize chromosomes as well as oat genome are important in photosynthetic efficiency.

Our study on morphological features and yielding of oat confirmed the high rank of cv. Bingo, which was used for producing all tested DH and OMA lines. Cv. Bingo, as a model cultivar, was characterized by the longest flag leaf, the greatest production of lateral shoots, and the highest TGW. In turn, among the tested lines, OMA ~~II~~ also deserves attention, which, apart from the wider flag leaf, the tallest main shoot, and the longest panicle, had a TGW comparable to cv. Bingo. Similar observation was described by Warzecha et al. [18], where two OMA lines (9 and 78b) had high yielding potential, comparable with commercial cv. Bingo under optimal soil hydration. Furthermore, the above mentioned lines had better yielding potential also under soil drought stress, which was reflected in a significantly lower reduction in the number and mass of grains per plant. Also, the authors found that the differences in both morphology and the number of seeds between tested lines appear to result more from the parental crosses than the introgression of maize chromosomes.

~~According to all available data,~~ the evolutionary transition from C_3 ancestors to C_4 plants took place repeatedly and independently in a variety of taxonomically diverse groups [32,33]. This could have led to a number of intermediates that were either matched or unmatched between anatomical features and physiological traits. Since already a few independent reversals from C_4

to C_3 have been reported [34], therefore, it seems probable that the ancestor of the Pooideae, to which ~~belong~~ oat, barley, and wheat~~,~~ underwent a C_4 to C_3 reversal early in its evolution [35].

Conclusions

The hypothesis that the introgression of maize chromosomes into the oat genome may cause changes in the leaf anatomy, plant morphology, photosynthetic efficiency, and yield was confirmed with regard to the following morphological features: flag leaf width, height of main shoot, panicle length, and weight of a thousand grains per plant in OMA II, especially when comparing with oat cv. Bingo. The introgression of maize chromosomes into the oat genome did not show comprehensive changes in leaf anatomy consistent with C4 photosynthesis, but in the OMA II line the big cells of the outer bundle sheath and their size of about 500 μm^2 was similar to the size of these cells in maize. These data show that maize chromosomes are capable of modifying the size of the bundle sheath cells, the trait associated with C4 photosynthesis, but this is definitely dependent on the oat genome.

Materials and Methods

Plant material and growth conditions

The objects of our study were oat plants (*Avena sativa* L.): *cv*. Bingo; two DH lines (Flamingstern × Bingo and STH 9787(b) × Bingo); and two OMA lines (Flamingstern × Bingo and STH 9787(b) × Bingo) with the same number of maize chromosomes added to oat genome. Oat DH lines were obtained through oat × maize crosses according to the method described by Warchoł et al. [36]. Oat seeds were derived from Strzelce Plant Breeding Ltd., Małopolska Plant Breeding Polanowice Ltd. and Danko Plant Breeding Ltd. The day after pollinating emasculated oat flowers by maize, one drop of 100 mg dm^{-3} 2,4-dichlorophenoxyacetic acid (2,4-D) was applied to each oat pistil. Developed embryos were isolated from ovaries 21 days

after pollination and cultured on 190-2 medium [37], after which developed haploid plants were transferred on MS medium [38]. The embryos germinated at 21 ± 2 °C and light intensity equal to 60 µmol m^{-2} s^{-1} and 16/8 h light/dark cycle. Haploid plants were acclimated to ex vitro conditions by transferring them to a moist perlite and then to the soil at 20 ± 2 °C and light intensity 110 µmol m^{-2} s^{-1}. After colchicine treatment, the oat plants were grown in a greenhouse in natural (solar) light intensity of 800 µmol m^{-2} s^{-1} during the day and sodium lamps between 6-8 a.m., 6-10 p.m., and on cloudy days at 21-28/17 °C day/night.

Identification of oat × maize hybrids

DNA extraction (Genomic Mini AX Plant Kit, A&A Biotechnology, Gdynia, Poland) and PCR analyses (2720 Thermal Cycler; Applied Biosystems, Foster City, CA, USA) were performed according to Skrzypek et al. [21]. Two Grande-1F (50-AAA GAC CTC ACG AAA GGC CCA AGG-30), Grande-1R (50-AAA TGG TTC ATG CCG ATT GCA CG-30) primers (GenBank accession number X97604 [39]) were used for the PCR reaction, which in successive cycles enabled the amplification of the 500 bp retrotransposon region of *Grande-1* and detection of the presence of maize chromatin in oat plants. The obtained products were separated in 1.5% agarose gel with ethidium bromide (Sigma-Aldrich, St. Louis, MO, USA) in TBE buffer, under 90 V for 90 min. DNA markers of 100 bp to 1000 bp and concentration of 0.5 mg/ml (GeneRuler 100bp; Fermentas, Waltham, MA, USA) were used to estimate the length of PCR products. The image of electrophoretic separation was archived using the Imagemaster VDS gel reader (Amersham, Pharmacia Biotech, Piscataway, NJ, USA) and the Liscap Capture Application ver. 1.0. Electrophoretic gel analysis was performed using GelScan ver. 1.45 (Kucharczyk Electrophoretic Techniques, Warsaw, Poland). The plants identified as having a *Grande-1* retrotransposon fragment were used for genomic in situ hybridization (GISH).

Mitotic chromosome preparations

Cytogenetic preparations were made according to the procedure described by Jenkins and Hasterok [40]. The root tips were excised from the 3-day-old seedlings, incubated in ice-cold water for 24 h and fixed in Carnoy's fixative (methanol and glacial acetic acid at a 3:1 ratio). The material was stored at -20 °C. To make cytogenetic preparations, the root meristems were washed in 0.01 M citrate buffer (pH 4.8) for 3 × 5 minutes, digested in an enzyme mixture containing 6% pectinase (v/v, Sigma), 1% cellulase (w/v, Sigma) and 1% cellulase 'Onozuka R-10'(w/v, Serva, Heidelberg, Germany) in citrate buffer at 37 °C for 2 h. The root tips were washed again in the citrate buffer. The meristems dissected from the root tips were placed in a drop of 45% acetic acid on a microscopic slide, covered with a coverslip, and gently squashed. Slides were incubated on dry ice to remove the coverslips and then stored at 4 °C until used.

Probe labeling and genomic in situ hybridization

In order to discriminate maize chromosomes in oat genomic background, the maize cv 'Waza' total genomic DNA (gDNA) was used as a probe for genomic in situ hybridization (GISH). Maize gDNA was extracted from young leaves using standard C-TAB procedure and labeled with digoxygenin-11-dUTP (Roche, Basel, Switzerland) by nick-translation method according to manufacturer instructions.

GISH procedure followed the protocol described in Skrzypek et al. [21]. Briefly, the slides were pre-treated with RNase (100 μg/mL) in 2 × saline sodium citrate (SSC) buffer for 1 h, washed 3 × 5 min in 2 × SSC, post-fixed in 1% formaldehyde, washed again in 2 × SSC, and dehydrated in ethanol series (70%, 90%, 100%). The labeled maize gDNA was precipitated and dissolved in a hybridization mixture containing 50% deionized formamide, 10% dextran sulfate, 2 × SSC, 0.5% SDS, and water. The hybridization mixture was predenatured (75 °C, 10 min), applied to air-dried slides, and denatured again at 75 °C for 4.5 min. Hybridization was carried out in a

humid chamber for about 40 hours at 37 °C. Post-hybridization washes were performed in 10% formamide in 0.1 × SSC at 42 °C. The hybridization signals were detected by antidigoxigenin fluorescein-conjugated antibodies (Roche, Basel, Switzerland). The chromosomes were mounted and counterstained in VectaShield Antifade (Vector Laboratories, Burlingame, CA, USA) containing 2.5 mg/mL DAPI (Serva, Heidelberg, Germany). Hybridization signals were visualized and captured using fluorescence microscope Axio Imager Z2 (ZEISS, Germany) equipped with monochromatic camera AxioCam MRm (ZEISS, Oberkochen, Germany). The acquired images were digitally processed and superimposed using ZEN blue program (ZEISS, Oberkochen, Germany) and Photoshop CS3 (Adobe, San Jose, CA, USA).

Maize chromosome identification

For maize chromosome identification, the total genomic DNA of OMA I and OMA II lines, oat *cv*. Bingo, and maize *cv*. Waza was extracted from ca. 0.8 g of fresh tissue frozen with liquid nitrogen using DNeasy Plant Mini Kit (Qiagen, Hilden, Germany). The added maize chromosomes in the tested OMA lines were identified by PCR with the simple sequence repeat (SSR) markers specific for particular maize chromosomes. Suitable SSR markers were chosen from the Maize Genome Database (https://www.maizegdb.org) and described before by Kynast et al. [8].

Anatomical analysis of the leaves

To examine the anatomical arrangements, leaf material was harvested from the widest points of the fully expanded fourth leaves and prepared for light microscopy. Images of leaves' transverse cross-sections were carried out using the paraffin method as described by Ruzin [41]. Samples were fixed in 50% ethanol and next dehydrated in a graded series of ethanol. The tissues embedded in paraffin (Histowax, 56-58°C) were cut using a sliding microtome (Leica SM 2000 R).The 5-7 μm thick sections were mounted on glass slides and double-stained with

safranin (1%, w/v) and alcian blue (1%, w/v) then mounted in Canada balsam. The cross-sections were observed with the light microscope Leica DM2000 and photographed with the digital camera DFC320. The following morphometric analyses were made by using the IM 1000 software package: distance between two small vascular bundles (Fig 5A); total area of mesophyll (area of chlorenchyma within the leaf) between two small vascular bundles (Fig 5B); number of cells in inner bundle sheath (first ring of cells surrounding one small vascular bundle) (Fig 5C); number of cells in outer bundle sheath (second ring of cells surrounding one small vascular bundle) (Fig 5C); area of inner bundle sheath (total area of all cells); and area of outer bundle sheath (total area of all cells). The average size of a single cell in the inner bundle sheath was calculated based on the data of the area of the inner bundle sheath and the number of cells in the inner bundle sheath. The same procedure was done for the outer bundle sheath.

Fig 5. Morphometric analyses of oat (*Avena sativa* L.) leaf made by using the IM 1000 software package: (A) distance between two small vascular bundles; (B) total area of mesophyll (area of chlorenchyma within the leaf) between two small vascular bundles; (C) number of cells in inner bundle sheath (first ring of cells which surround one small vascular bundle – blue color) and number of cells in outer bundle sheath (second ring of cells which surround one small vascular bundle – red color).

Chlorophyll a fluorescence kinetics parameters

The measurements of (PCF) were conducted using a Handy PEA fluorometer (Hansatech, Kings Lynn, UK). The measurements were carried out on the flag leaves as described by Juzoń et al. 2020 [17]. The leaves' fragments were shaded with clips for about 20 minutes and then the measurement was taken. It was initiated by illuminating the leaf fragment with saturated light (intensity of 90 units = 5,400 μmol (quantum) $m^{-2}s^{-1}$, illumination time 0.9 s) to determine the maximum fluorescence (F_m) and calculate the maximum photochemical potential (F_v/F_m).

Then, actinic light was switched on automatically (25 units = 1,500 µmol (quantum) $m^{-2}s^{-1}$) for a period of 270 s. When it was switched off, the leaf light was emitted by a diode emitting light in the far red range of about 15 W m^{-2}. On the basis of chlorophyll *a* fluorescence measurements, the following parameters were calculated and analyzed: F_v/F_m – maximum photochemical efficiency of PSII; Area – area over the chlorophyll *a* fluorescence induction curve; PI – overall performance index of PSII photochemistry; ABS/CS – light energy absorption; TR_0/CS – excitation energy trapped in PSII reaction centers; ET_0/CS – energy used for electron transport; DI_0/CS – energy dissipated from PSII; and RC/CS_0 – number of active reaction centers in the excited leaf fragment.

Yield component analyses

To estimate the impact of maize chromosomes added to oat genome, morphological observations (flag leaf length and width, height of main shoot, panicle length) were carried out and selected yield components (number of shoots/plant, number and weight of grains/plant, thousand grain weight (TGW)) were assessed.

Statistical analysis

The results presented in the Figures constitute the mean values based on five plants as replicates. In each plant, the measurements were performed on five leaves. Data were analyzed using STATISTICA v. 13 (Stat-Soft, USA) software. One-way analysis of variance and Duncan's multiple range test at the 0.05 probability level was used to determine the significance of differences between lines/cultivar, which was marked with different letters.

Acknowledgements

Radenko Radošević for technical helping of leaves' transverse cross-sections preparation.

References

1. Laurie DA, Bennett MD. The timing of chromosome elimination in hexaploid wheat × maize crosses. Genome. 1989;32: 953–961. doi: 10.1139/g89-537

2. Rines HW, Dahleen LS. Haploid Oat Plants Produced by Application of Maize Pollen to Emasculated Oat Florets. Crop Sci. 1990;30: 1073-1078. doi: 10.2135/cropsci1990.0011183X003000050023x

3. Houben A, Sanei M, Pickering R. Barley doubled-haploid production by uniparental chromosome elimination. Plant Cell, Tissue Organ Cult. 2011;104: 321–327. doi: 10.1007/s11240-010-9856-8

4. Laurie DA, Bennett MD. Cytological Evidence for Fertilization in Hexaploid Wheat × Sorghum Crosses. Plant Breed. 1988;100: 73–82. doi: 10.1111/j.1439-0523.1988.tb00220.x

5. Riera-Lizarazu O, Rines HW, Phillips RL. Cytological and molecular characterization of oat × maize partial hybrids. Theor Appl Genet. 1996;93: 123–135. doi: 10.1007/BF00225737

6. Mochida K, Tsujimoto H, Sasakuma T. Confocal analysis of chromosome behavior in wheat × maize zygotes. Genome. 2004;47: 199–205. doi: 10.1139/g03-123

7. Ishii T, Ueda T, Tanaka H, Tsujimoto H. Chromosome elimination by wide hybridization between Triticeae or oat plant and pearl millet: pearl millet chromosome dynamics in hybrid embryo cells. Chromosom Res. 2010;18: 821–831. doi: 10.1007/s10577-010-9158-3

8. Kynast RG, Riera-Lizarazu O, Vales MI, Okagaki RJ, Maquieira SB, Chen G, et al. A Complete Set of Maize Individual Chromosome Additions to the Oat Genome. Plant Physiol. 2001;125: 1216–1227. doi: 10.1104/pp.125.3.1216

9. Okagaki RJ, Kynast RG, Livingston SM, Russell CD, Rines HW, Phillips RL. Mapping Maize Sequences to Chromosomes Using Oat-Maize Chromosome Addition Materials,. Plant Physiol. 2001;125: 1228–1235. doi: 10.1104/pp.125.3.1228

10. Jin W, Melo JR, Nagaki K, Talbert PB, Henikoff S, Dawe RK, et al. Maize Centromeres: Organization and Functional Adaptation in the Genetic Background of Oat. Plant Cell. 2004;16: 571–581. doi: 10.1105/tpc.018937

11. Walch MD. Expression of maize pathogenesis-related and photosynthetic genes in oat × maize addition lines. M. Sc. Thesis, University of Minnesota. 2007.

12. Kowles R V., Walch MD, Minnerath JM, Bernacchi CJ, Stec AO, Rines HW, et al. Expression of C_4 photosynthetic enzymes in oat-maize chromosome addition lines. Maydica. 2008;53: 69–78.

13. Dengler NG, Nelson T. Leaf Structure and Development in C_4 Plants. In: Sage RS, Monson RK, editors. C_4 Plant Biology. San Diego, CA, USA: Academic Press; 1999. pp. 133–172.

14. Sage RF, Khoshravesh R, Sage TL. From proto-Kranz to C_4 Kranz: building the bridge to C_4 photosynthesis. J Exp Bot. 2014;65: 3341–3356. doi: 10.1093/jxb/eru180

15. Tolley BJ, Sage TL, Langdale JA, Hibberd JM. Individual maize chromosomes in the

C_3 plant oat can increase bundle sheath cell size and vein density. Plant Physiol. 2012;159: 1418–1427. doi: 10.1104/pp.112.200584

16. Williams BP, Johnston IG, Covshoff S, Hibberd JM. Phenotypic landscape inference reveals multiple evolutionary paths to C_4 photosynthesis. Elife. 2013;2: e00961. doi: 10.7554/eLife.00961.001

17. Juzoń K, Idziak-Helmcke D, Rojek-Jelonek M, Warzecha T, Warchoł M, Czyczyło-Mysza I, et al. Functioning of the Photosynthetic Apparatus in Response to Drought Stress in Oat × Maize Addition Lines. Int J Mol Sci. 2020;21: 6958. doi: 10.3390/ijms21186958

18. Warzecha T, Bathelt R, Skrzypek E, Warchoł M, Bocianowski J, Sutkowska A. Studies of Oat-Maize Hybrids Tolerance to Soil Drought Stress. Agriculture. 2023;13: 243. doi: 10.3390/agriculture13020243

19. Kajala K, Covshoff S, Karki S, Woodfield H, Tolley BJ, Dionora MJA, et al. Strategies for engineering a two-celled C_4 photosynthetic pathway into rice. J Exp Bot. 2011;62: 3001–3010. doi: 10.1093/jxb/err022

20. Cabral CB. Expression of maize genes on a specific chromosome in the presence of a foreign genome. PhD Thesis. University of Minnesota. 2009. Available from: https://conservancy.umn.edu/bitstream/handle/11299/56590/1/Cabral_umn_0130E_10629.pdf

21. Skrzypek E, Warzecha T, Noga A, Warchoł M, Czyczyło-Mysza I, Dziurka K, et al. Complex characterization of oat (*Avena sativa* L.) lines obtained by wide crossing with maize (*Zea mays* L.). PeerJ. 2018;6: e5107. doi: 10.7717/peerj.5107

22. Song Q, Chen ZJ. Epigenetic and developmental regulation in plant polyploids. Curr Opin Plant Biol. 2015;24: 101–109. doi: 10.1016/j.pbi.2015.02.007

23. Ravi M, Chan SWL. Haploid plants produced by centromere-mediated genome elimination. Nature. 2010;464: 615–618. doi: 10.1038/nature08842

24. Phillips RL, Rines HW. Genetic Analyses with Oat-Maize Addition and Radiation Hybrid Lines. In: Bennetzen JL, Hake S, editors. Handbook of Maize. New York, NY, USA: Springer; 2009. pp. 523–538.

25. Dong Z, Yu J, Li H, Huang W, Xu L, Zhao Y, et al. Transcriptional and epigenetic adaptation of maize chromosomes in Oat-Maize addition lines. Nucleic Acids Res. 2018;46: 5012–5028. doi: 10.1093/nar/gky209

26. Cho S, Garvin DF, Muehlbauer GJ. Transcriptome Analysis and Physical Mapping of Barley Genes in Wheat–Barley Chromosome Addition Lines. Genetics. 2006;172: 1277–1285. doi: 10.1534/genetics.105.049908

27. Brown HR, Bouton JH. Physiology and Genetics of Interspecific Hybrids Between Photosynthetic Types. Annu Rev Plant Physiol Plant Mol Biol. 1993;44: 435–456. doi: 10.1146/annurev.pp.44.060193.002251

28. Ueni O, Sentoku N. Comparison of leaf structure and photosynthetic characteristics of C_3 and C_4 *Alloteropsis semialata* subspecies. Plant, Cell Environ. 2006;29: 257–268. doi: 10.1111/j.1365-3040.2005.01418.x

29. Dengler NG, Dengler RE, Donnelly PM, Hattersley PW. Quantitative Leaf Anatomy of

C_3 and C_4 Grasses (Poaceae): Bundle Sheath and Mesophyll Surface Area Relationships. Ann Bot. 1994;73: 241–255. doi: 10.1006/anbo.1994.1029

30. Hibberd JM, Sheehy JE, Langdale JA. Using C_4 photosynthesis to increase the yield of rice—rationale and feasibility. Curr Opin Plant Biol. 2008;11: 228–231. doi: 10.1016/j.pbi.2007.11.002

31. Sage RF, Zhu X-G. Exploiting the engine of C_4 photosynthesis. J Exp Bot. 2011;62: 2989–3000. doi: 10.1093/jxb/err179

32. Sage TL, Busch FA, Johnson DC, Friesen PC, Stinson CR, Stata M, et al. Initial Events during the Evolution of C_4 Photosynthesis in C_3 Species of Flaveria. Plant Physiol. 2013;163: 1266–1276. doi: 10.1104/pp.113.221119

33. Lundgren MR, Dunning LT, Olofsson JK, Moreno-Villena JJ, Bouvier JW, Sage TL, et al. C_4 anatomy can evolve via a single developmental change. Ecol Lett. 2019;22: 302–312. doi: 10.1111/ele.13191

34. Vicentini A, Barber JC, Aliscioni SS, Giussani LM, Kellogg EA. The age of the grasses and clusters of origins of C_4 photosynthesis. Glob Chang Biol. 2008;14: 2963–2977. doi: 10.1111/j.1365-2486.2008.01688.x

35. Slewinski TL. Using evolution as a guide to engineer kranz-type C_4 photosynthesis. Front Plant Sci. 2013;4: 212. doi: 10.3389/fpls.2013.00212

36. Warchoł M, Skrzypek E, Nowakowska A, Marcińska I, Czyczyło-Mysza I, Dziurka K, et al. The effect of auxin and genotype on the production of *Avena sativa* L. doubled haploid lines. Plant Growth Regul. 2016;78: 155–165. doi: 10.1007/s10725-015-0082-6

37. Zhuang JJ, Xu J. Increasing differentiation frequencies in wheat pollen callus. In: Hu H, Vega M, editors. Cell and tissue culture techniques for cereal crop improvement. Benjing, China: Science Press; 1983. pp. 431.

38. Murashige T, Skoog F. A revised medium for rapid growth and bio assays with tobacco tissue cultures. Physiol Plant. 1962;15: 473–497. doi: 10.1111/j.1399-3054.1962.tb08052.x

39. Ananiev E V., Phillips RL, Rines HW. Chromosome-specific molecular organization of maize (*Zea mays* L.) centromeric regions. Proc Natl Acad Sci. 1998;95: 13073–13078. doi: 10.1073/pnas.95.22.13073

40. Jenkins G, Hasterok R. BAC ‘landing’ on chromosomes of *Brachypodium distachyon* for comparative genome alignment. Nat Protoc. 2007;2: 88–98. doi: 10.1038/nprot.2006.490

41. Ruzin SE. Plant microtechnique and microscopy. New York, NY, USA: Oxford University Press; 1999.

Author Contributions

Conceptualization, M.W., E.S., K.J-S. and D.R.; methodology, M.W., E.S., D.R., I.P., T.W., and D.I-H; formal analysis, M.W., D.R., and K.J-S.; investigation, E.S., I.Cz-M., K.D., D.R., I.P., K.J-S., T.W., and K.L.; resources, M.W., D.R; writing – original draft preparation, M.W., E.S., and K.J-S.; writing – review and editing, D.R., I.P., T.W., D.I-H., and K.L.; visualization, E.S., D.I-H., T.W., and D.R.; supervision, M.W.; project administration, E.S. All authors have read and agreed to the published version of the manuscript.

Figure 1

Click here to access/download;Figure;Fig1.tif.tif

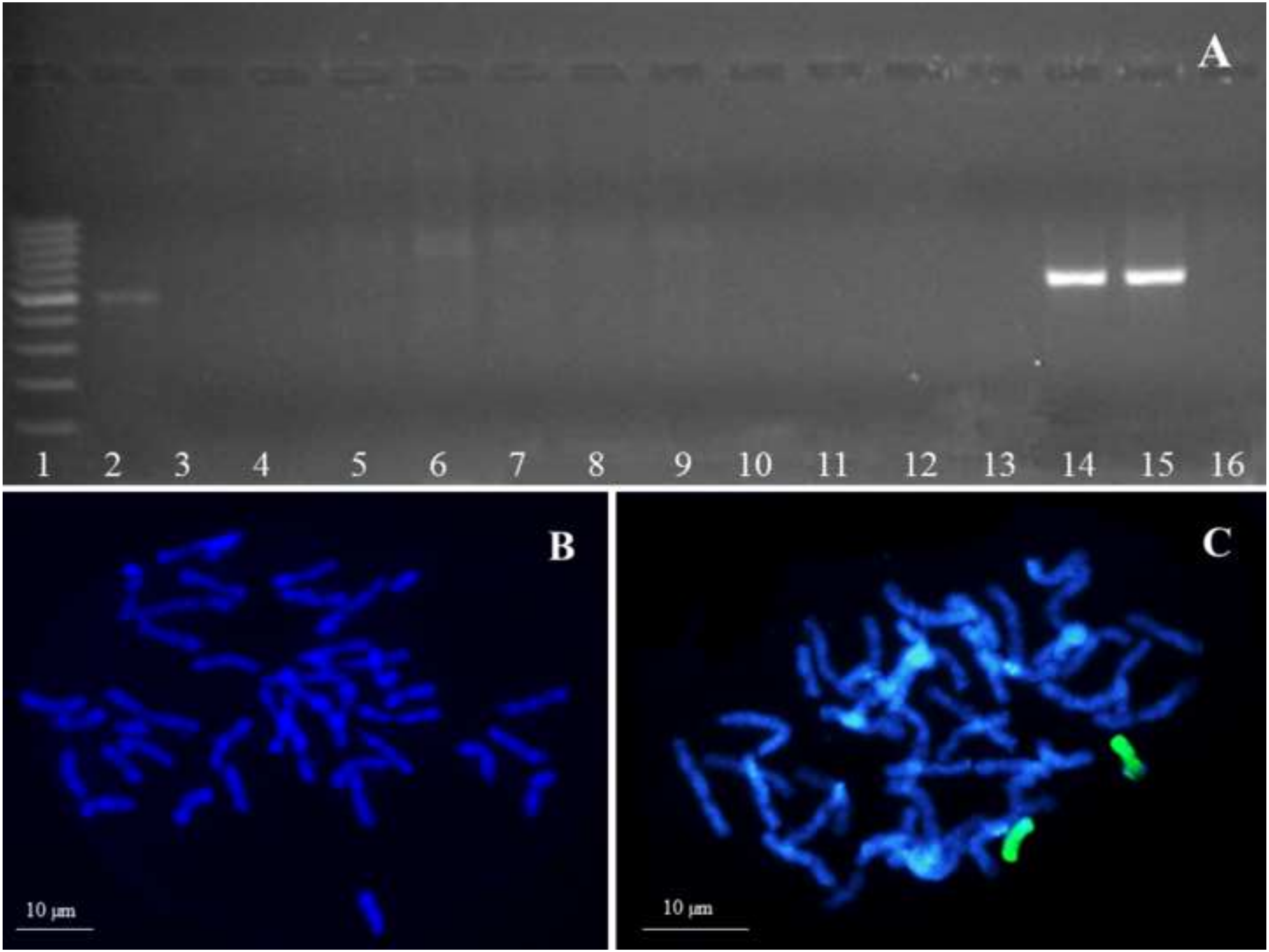

Figure 2

Click here to access/download;Figure;Fig2.tif.tif

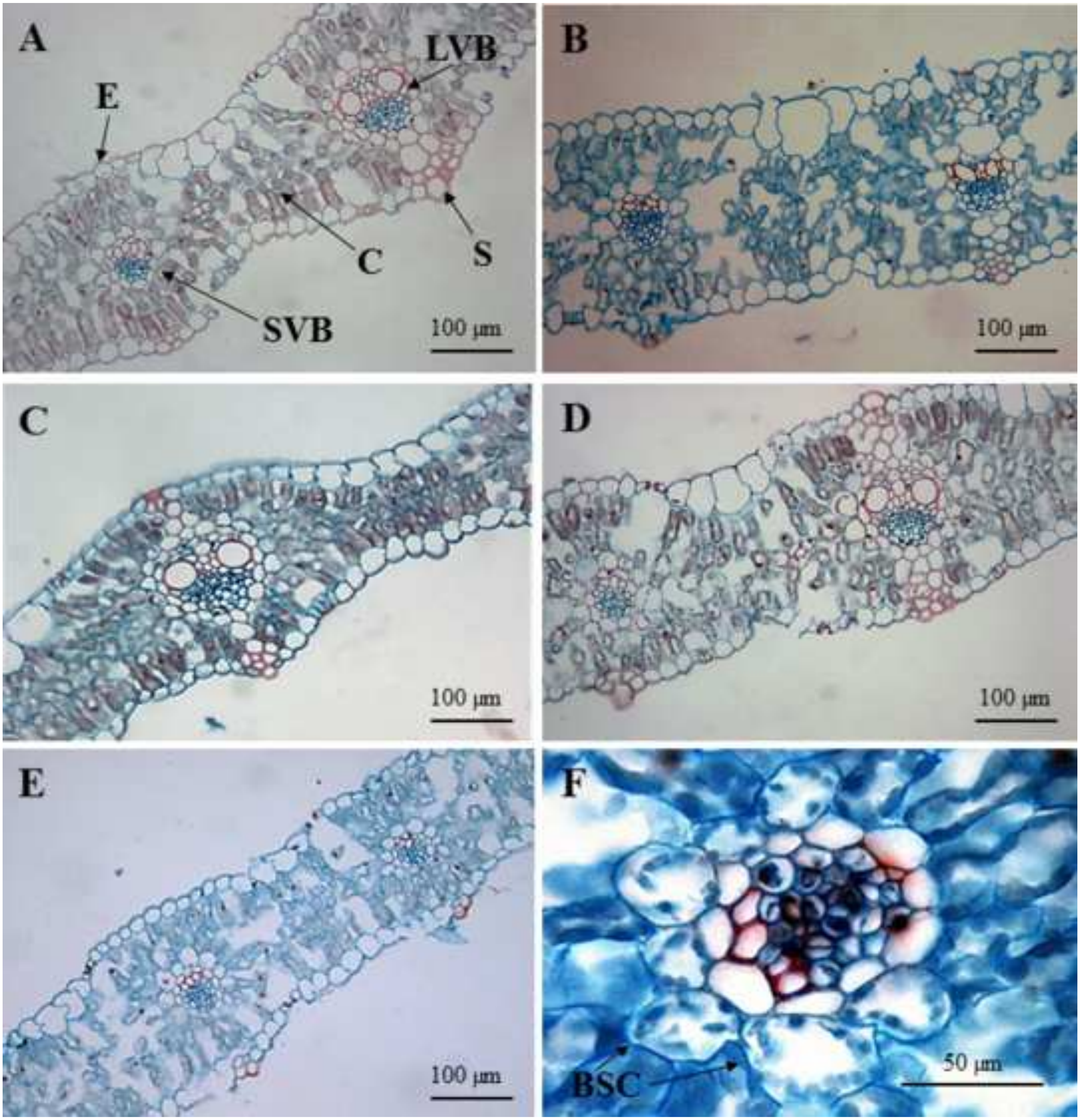

Figure 3

Click here to access/download;Figure;Fig3.tif.tif

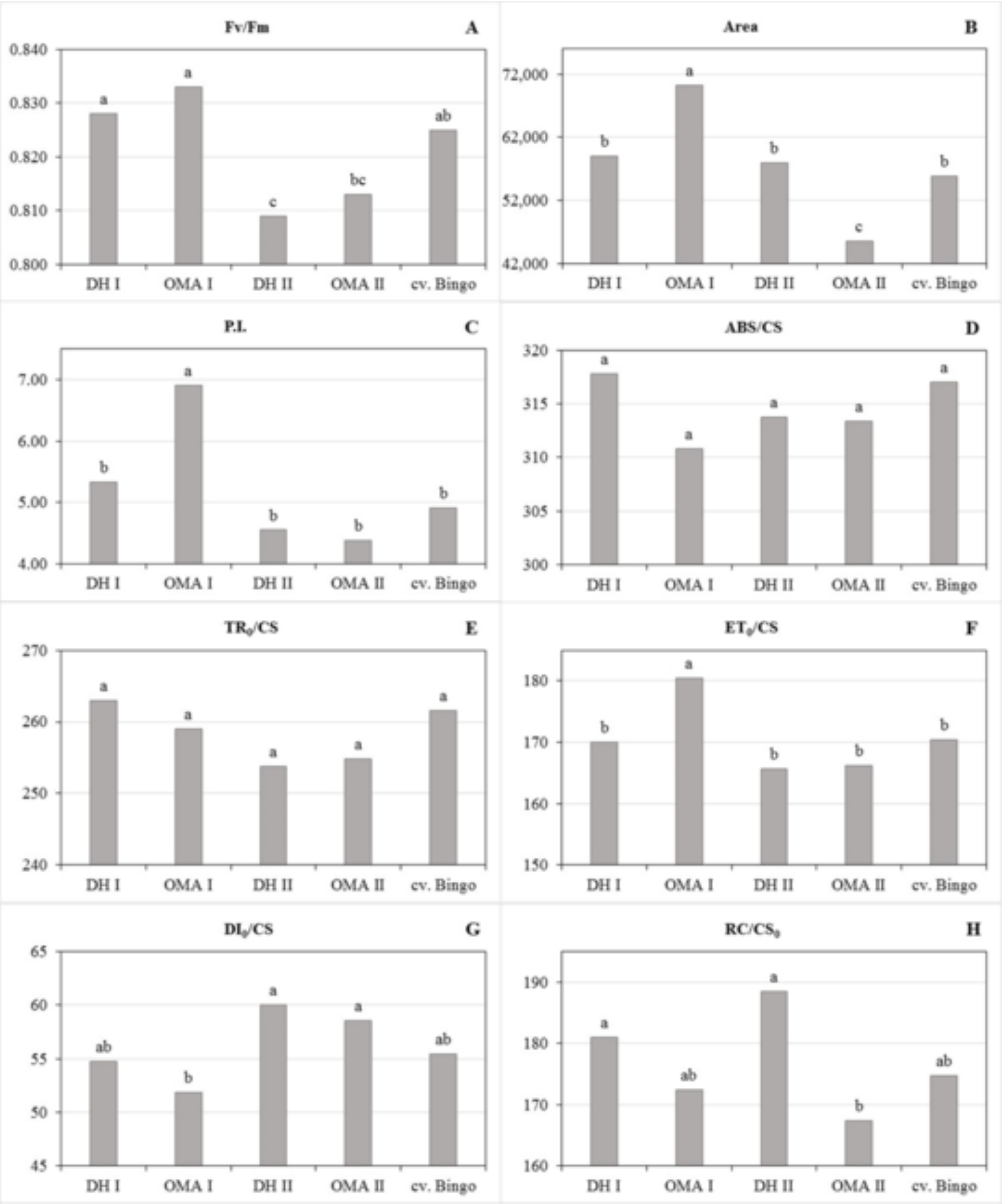

Figure 4 Click here to access/download;Figure;Fig4.tif.tif

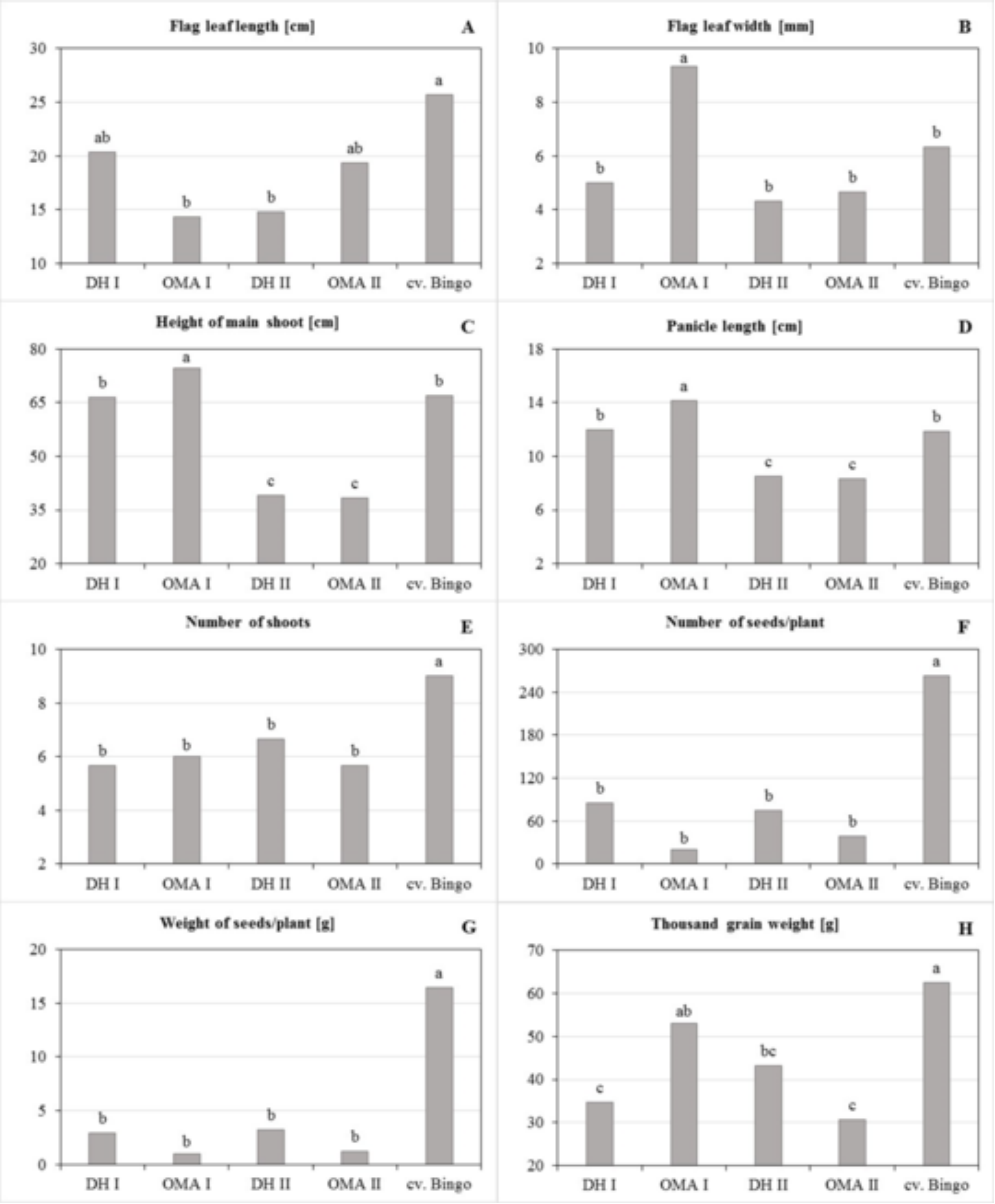

Figure 5

Click here to access/download;Figure;Fig5.tif.tif

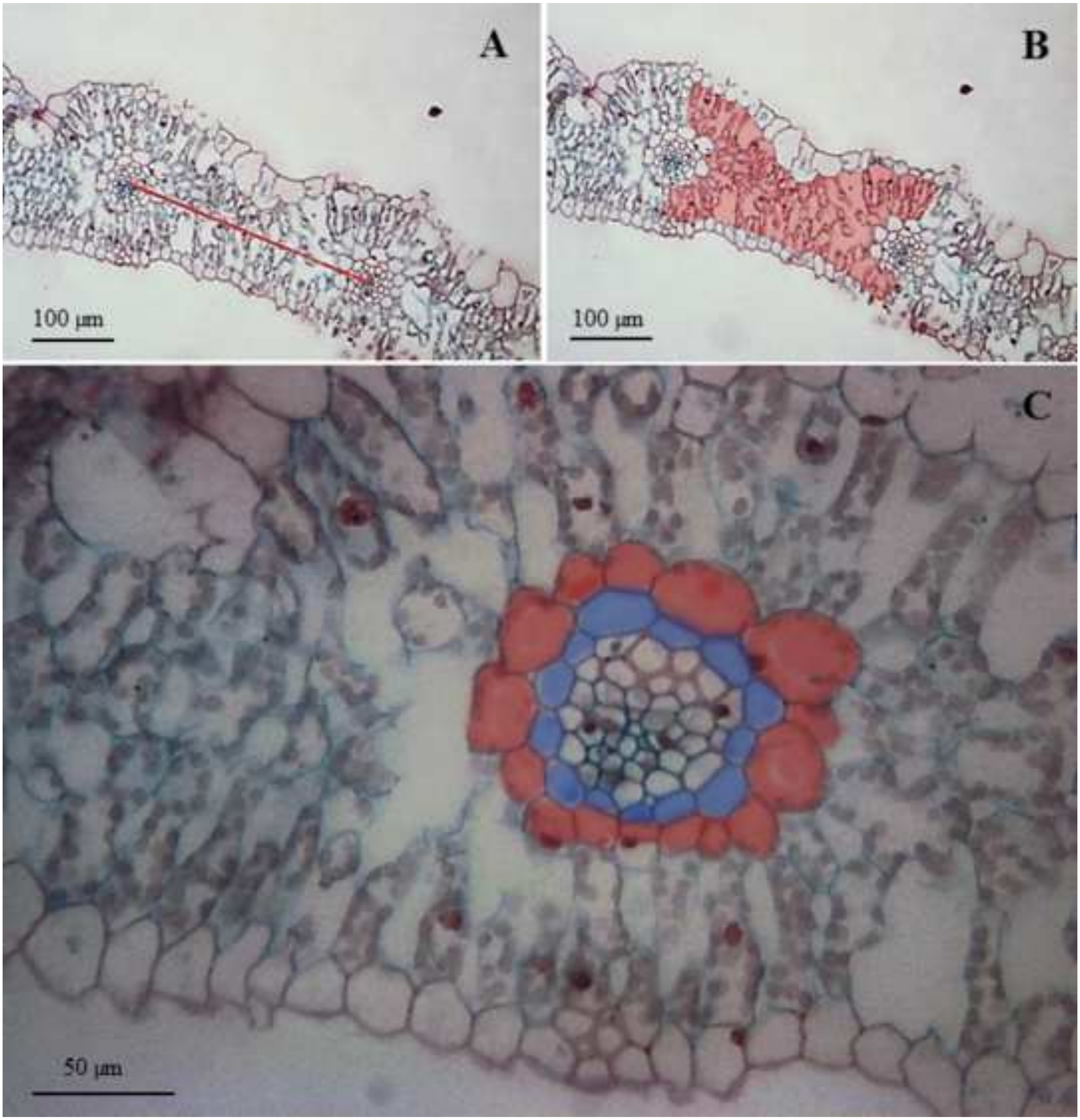

Supplement: Attachment — Submitted filename: PONE-D-23-25096_reviewer.pdf [file pone.0298072.s003.pdf]
