# Supplementary material for: Comparative characteristics of oat doubled haploids and oat × maize addition lines: Anatomical features of the leaves, chlorophyll a fluorescence and yield parameters
Source: PLoS One. 2024 Apr 9;19(4):e0298072. doi: 10.1371/journal.pone.0298072 (PMC11003612; doi:10.1371/journal.pone.0298072)
Supplement: Attachment — Submitted filename: Response to Reviewers_12.10.23.docx [file pone.0298072.s004.docx]

**Dear Editor,**

**we would like to express our gratitude to the Editor and Reviewers for comments and critics which encourage us to improve our manuscript, especially statistical methods. Please find below our response to Reviewers’ comments since we would like to clarify some issues concerned by Reviewers.**

Journal Requirements:

When submitting your revision, we need you to address these additional requirements.

1. Please ensure that your manuscript meets PLOS ONE's style requirements, including those for file naming. The PLOS ONE style templates can be found at

https://journals.plos.org/plosone/s/file?id=wjVg/PLOSOne_formatting_sample_main_body.pdf

**Answer: We checked the requirements to conform the manuscript to proper form.**

2. We noticed you have some minor occurrence of overlapping text with the following previous publication(s), which needs to be addressed:

https://www.mdpi.com/2077-0472/13/2/243

https://www.ncbi.nlm.nih.gov/pmc/articles/PMC2948986/

In your revision ensure you cite all your sources (including your own works), and quote or rephrase any duplicated text outside the methods section. Further consideration is dependent on these concerns being addressed.

**Answer: We rephrased duplicated text from Warzecha et al. 2023 (**[**https://www.mdpi.com/2077-0472/13/2/243**](https://www.mdpi.com/2077-0472/13/2/243)**).**

**From Wesrhoff and Gowik 2010 (**[**https://www.ncbi.nlm.nih.gov/pmc/articles/PMC2948986**](https://www.ncbi.nlm.nih.gov/pmc/articles/PMC2948986)**), we rephrased part about density of vein spacing in Kranz anatomy, and additionally we have corrected the error in the quoted literature. In our manuscript we cited Ueno and Sentoku (2006), whereas there should be Ueno et al. (2006).**

3. PLOS ONE now requires that authors provide the original uncropped and unadjusted images underlying all blot or gel results reported in a submission’s figures or Supporting Information files. This policy and the journal’s other requirements for blot/gel reporting and figure preparation are described in detail at https://journals.plos.org/plosone/s/figures#loc-blot-and-gel-reporting-requirements and https://journals.plos.org/plosone/s/figures#loc-preparing-figures-from-image-files. When you submit your revised manuscript, please ensure that your figures adhere fully to these guidelines and provide the original underlying images for all blot or gel data reported in your submission. See the following link for instructions on providing the original image data: https://journals.plos.org/plosone/s/figures#loc-original-images-for-blots-and-gels.
 
In your cover letter, please note whether your blot/gel image data are in Supporting Information or posted at a public data repository, provide the repository URL if relevant, and provide specific details as to which raw blot/gel images, if any, are not available. Email us at plosone@plos.org if you have any questions.

**Answer: We added the original gel image to Supporting Information (S1_Fig), one-line title and information in our manuscript.**

**S1 Fig. Original, uncropped electrophoresis gel picture underlying Fig 1 from the main text.**

4. Please review your reference list to ensure that it is complete and correct. If you have cited papers that have been retracted, please include the rationale for doing so in the manuscript text, or remove these references and replace them with relevant current references. Any changes to the reference list should be mentioned in the rebuttal letter that accompanies your revised manuscript. If you need to cite a retracted article, indicate the article’s retracted status in the References list and also include a citation and full reference for the retraction notice.

**Answer: We checked the reference list and corrected citation in the text.**

**We retracted one reference: Ueni O, Sentoku N. Comparison of leaf structure and photosynthetic characteristics of C3 and C4 Alloteropsis semialata subspecies. Plant, Cell Environ. 2006;29: 257–268. doi: 10.1111/j.1365-3040.2005.01418.x**

**We added 3 references:**

**Ueno O, Kawano Y, Wakayama M, Takeda T. Leaf vascular systems in C3 and C4 grasses: a two-dimensional analysis. Ann Bot. 2006; 97: 611–621. doi: 10.1093/aob/mcl010**

**Ishii T., Tanaka H., Eltayeb A.E., Tsujimoto H. (2013) Wide hybridization between oat and pearl millet belonging to different subfamilies of Poaceae. Plant Reprod., 26:25–32**

**Warzecha, T.; Bocianowski, J.; Warchoł, M.; Bathelt, R.; Sutkowska, A.; Skrzypek, E. Effect of Soil Drought Stress on Selected Biochemical Parameters and Yield of Oat × Maize Addition (OMA) Lines. Int. J. Mol. Sci. 2023, 24, 13905. https://doi.org/10.3390/ijms241813905**

Reviewers' comments:

5. Review Comments to the Author

Please use the space provided to explain your answers to the questions above. You may also include additional comments for the author, including concerns about dual publication, research ethics, or publication ethics. (**Please upload your review as an attachment if it exceeds 20,000 characters**)

**Reviewer #1**: Manuscript "Comparative characteristics of oat doubled haploids and oat × maize addition lines: anatomical features of the leaves, chlorophyll a fluorescence and yield parameters" is very interesting.

General comments:
Authors evaluated how the presence of maize chromosomes changes the anatomical parameters of the leaves and functioning of photosynthetic apparatus of the disomic OMA lines compared to the DH lines and Bingo cultivar. Authors assessed also yield components for all lines.

Detailed comments:
**R1:** The introduction is quite interesting. However, most of the references are very old.

**Answer: Best to our knowledge, we cited all previously published references on the characteristics of OMA lines in relation to anatomical studies, kinetics of chlorophyll *a* fluorescence and their yield.**

**However, we have added very recent information to the introduction regarding this topic published by: Warzecha, T.; Bocianowski, J.; Warchoł, M.; Bathelt, R.; Sutkowska, A.; Skrzypek, E. Effect of Soil Drought Stress on Selected Biochemical Parameters and Yield of Oat × Maize Addition (OMA) Lines. Int. J. Mol. Sci. 2023, 24, 13905.** [**https://doi.org/10.3390/ijms241813905**](https://doi.org/10.3390/ijms241813905)

**R1:** Table 2 shows the results of the one-way ANOVA. I suggest supplementing is with mean squares for the difference factor and for the error. Then the table will be more readable.

**Answer: We corrected Table 2 as recommended by the reviewer. We added sum of squares, degrees of freedom, mean square and F-test values.**

**R1:** Table 3: The title says that the table should include SE. Unfortunately, these values are missing from the table. It should be completed.

**Answer: We completed Table 3 with SE values.**

**R1:** Table 4 shows the results of the one-way ANOVA. I suggest supplementing is with mean squares for the difference factor and for the error. Then the table will be more readable.

**Answer: We corrected Table 4 as recommended by the reviewer. We added sum of squares, degrees of freedom, mean square and F-test values.**

**R1:** Table 5 shows the results of the one-way ANOVA. I suggest supplementing is with mean squares for the difference factor and for the error. Then the table will be more readable.

**Answer: We corrected Table 5 as recommended by the reviewer. We added sum of squares, degrees of freedom, mean square and F-test values.**

**R1:** Figure 3: The caption says that the drawing should include SE. Unfortunately, these values are missing from the drawing. It should be completed.

**Answer: We completed Figure 3 with SE values.**

**R1:** Figure 4: The caption says that the drawing should include SE. Unfortunately, these values are missing from the drawing. It should be completed.

**Answer: We completed Figure 4 with SE values.**

**R1:** The description of statistical methods is cursory. Among other things, information about the empirical distribution and meeting the assumptions of analysis of variance is missing.

**Answer: We supplemented the paragraph with additional statistical methods: Pearson’s linear correlation coefficients and principal component analysis which were used while the manuscript was revised. We also change the structure of this paragraph.**

**R1:** My suggestions:
The Authors evaluated yield components for all lines. Unfortunately, they were not tempted to analyze the effect of yield components on yield. This is a very important aspect in breeding. Such an analysis would have increased the value of the manuscript. I suggest conducting such an analysis.

**Answer: We performed the Pearson’s linear correlation coefficients to find the connection among yield and certain yield elements and described it in the last part of Results.**

**R1:** In the Introduction, the authors wrote that, to the best of their knowledge, this is the first time that OMA and DH lines produced from the same parental crosses have been compared in terms of leaf anatomical characteristics. This would suggest a comparison between groups of OMA and DH lines. Unfortunately, such a comparison is missing from the manuscript. I suggest making such a comparison using, for example, contrast analysis.

**Answer: The comparison of leaf anatomical traits of DH and OMA lines and cv. Bingo is presented in Table 3, were we applied the Duncan’s multiple test to compare DH and OMA lines. We also did analysis which revealed that there were no correlations of leaf anatomical traits between tested plant material (OMA and DH lines) with chlorophyll *a* fluorescence parameters and yields components. So, we do not present in the manuscript this analysis since it didn’t show any correlations.**

**R1:** The manuscript provides a description of many features. Each was discussed separately. In plant breeding, it is very important to determine the relationship between traits. This is very important in the selection process. I suggest conducting a correlation analysis.

**Answer: Thank you for the suggestion. We performed the Pearson’s linear correlation coefficients to determine the relationship between traits and we described that in Results sections.**

**R1:** Depending on the existence or absence of trait correlation, the selection process in breeding proceeds in different ways. The results of multivariate analysis are undoubtedly facilitating selection decisions. Supplementing the manuscript with the results of the analysis of canonical variables, together with the estimation of Mahalanobis distances between the studied genotypes, will be a valuable enrichment of the conducted research.

**Answer: We did not performed the analysis of canonical variables, together with the estimation of Mahalanobis distances between the studied genotypes because we do not have the access to such software. Instead of that we did Pearson’s linear correlation coefficients and biplot based on first two principal components axes (PC1 and PC2) for chlorophyll a fluorescence parameters of oat cv. Bingo, OMA and DH lines, distribution of oat genotypes based on the chlorophyll fluorescence parameters, for yield components of oat cv. Bingo, OMA and DH lines and distribution of oat genotypes based on the yield components. Instead of canonical variables, together with the estimation of Mahalanobis distances we performed the analysis of data with multivariate approach with the application of PCA analysis.**

**Reviewer #2:**

**R2:** Dear Authors,
thank you for a lovely paper that neatly describes the study you have done. I enjoyed reading it. I have made some very minor language edits to the paper which I hope will contribute positively.
The file is attached for your use.

**Answer: Thank you for the language editing, we corrected the manuscript according to your suggestions.**

**R2:** This is ambiguous - the same number of chromosomes as in one or the same chromosome as in both have Chr9. the sentence could be clarified by saying "both OMA contained a single copy of maize chromosome 9"

**Answer: We would like to explain the question connected with numbers of maize chromosomes: “In M&M section Line 347 we wrote that ...'and two OMA lines (Flamingstern × Bingo 347 and STH 9787(b) × Bingo) with the same number of maize chromosomes added to oat genome”. It means that in both OMA lines we detected 2 chromosomes. Chromosomes identification we showed in Table 1, where OMA I have 2 copy of maize chromosome 5 and OMA II have 1 copy of maize chromosome 3 and 1 copy of maize chromosome 8. We added that information to the M&M section.**
